# Incidence and neutralizing antibody seroprevalence of influenza B virus in Egypt: Results of a community-based cohort study

**DOI:** 10.1371/journal.pone.0269321

**Published:** 2022-06-29

**Authors:** Noura M. Abo Shama, Sara H. Mahmoud, Ola Bagato, Elsayed Tarek AbdElsalam, Maha Alkhazindar, Ahmed Kandeil, Pamela P. McKenzie, Richard J. Webby, Mohamed A. Ali, Ghazi Kayali, Rabeh El-Shesheny

**Affiliations:** 1 Center of Scientific Excellence for Influenza Virus, Environmental Research Division, National Research Centre, Giza, Egypt; 2 Institute of Molecular Virology and Cell Biology, Friedrich-Loeffler-Institut, Federal Research Institute for Animal Health, Riems, Germany; 3 Department of Botany and Microbiology, Faculty of Science, Cairo University, Gamaa Street, Giza, Egypt; 4 Department of Infectious Diseases, St. Jude Children’s Research Hospital, Memphis, TN, United States of America; 5 Human Link, Dubai, UAE; Uppsala University, SWEDEN

## Abstract

Since 2000, two lineages of influenza B viruses, Victoria and Yamagata, have been circulating at similar frequencies worldwide. Little is known about the circulation of those viruses in Egypt. This study aims to describe the epidemiology of influenza B virus infections in Egypt, 2017–2019. This was performed through a household prospective cohort study on influenza infections among 2400 individuals from five villages. When a study participant had influenza like symptoms, a nasal swab and an oropharyngeal swab were obtained and tested by RT-PCR for influenza B infections. A serum sample was obtained from all participants annually to detect neutralizing antibodies using microneutralization assay. 9.1% of subjects were positive for influenza B viruses during season 2017–2018 mostly among preschoolers and 7.6% were positive during the season 2018–2019 with higher risk in females, potentially due to mothers being infected after contact with their children. The overall seroprevalence among the participants was 53.2% and 52.2% against the Victoria and Yamagata lineages respectively, the majority of seropositive participants were students. Multivariate analysis showed that age and having chronic diseases were the strongest predictors of infection. Our results show that both influenza B lineages circulated between 2017 and 2020 in Egypt almost in equal proportion. Encouraging the uptake of seasonal influenza vaccines is recommended.

## Introduction

Human influenza viruses are classified into three types A, B, and C based on different structural arrangements of the internal genes [1]. Influenza A Viruses (IAVs) cause high morbidity and mortality in humans and are well studied. Influenza B Viruses (IBVs) that are less studied, are an important pathogen among children [2,3]. Currently circulating IBVs include two lineages which are genetically and antigenically distinct, B/Victoria/2/87 (Victoria lineage) and B/Yamagata/16/88 (Yamagata lineage) [4,5]. The Victoria lineage dominated in the 1980s, then the Yamagata lineage took over in the 1990s. The two lineages have been detected globally with similar frequency since 2000 [6].

The clinical symptoms related to influenza B virus infection are comparable to that of influenza A infection. Several studies however have linked influenza B infections to severe symptoms. The symptoms of IBV are usually mild to moderate in healthy individuals including children. Compared to adults, young children with IBVs had a more severe disease [7]. Yearly, IBVs cause epidemics worldwide accounting in some years for more than 23% of human seasonal influenza infections [8,9].

As a result of the co-circulation of both lineages, the evolution of IBV differs explaining thereby the variability of seasonal outbreaks [10]. Recent studies have pointed to potential differences in the epidemiology of Victoria and Yamagata lineage viruses, with the average age of Victoria virus infected people is younger and transmission rate higher. The Victoria lineage is more likely to experience antigenic drift being under stronger positive selection pressure than the Yamagata lineage which is more conserved [10].

Some studies reported clinical and epidemiological differences of IBVs. Between 2009 and 2012, active surveillance in South Africa revealed that patients with IBVs outnumbered patients with IAVs and this may be due to infection with Human Immunodeficiency Virus (HIV); consequently, prevention through vaccination was recommended [11]. In addition, influenza viruses were detected in 22% of samples from patients with Influenza-Like Illness (ILI) during 2005–2014, out of which 24% were IBVs. It was observed that the cases with ILI were highly susceptible to the two IBVs lineages and the prevalence of B/Victoria was high especially in children and cases infected with HIV. They showed that, IBV lineages co-circulated in seasons of 2005–2014 except in 2013 and 2014 in South Africa [12].

The most effective strategy for preventing influenza and its complications is annual influenza vaccination. Seasonal vaccines are usually trivalent with one influenza B lineage included. Although there are only two lineages, the dominant lineage changes over time rendering the selection of the right influenza B virus strain for the vaccine extremely difficult. If the selected vaccine does not match the circulating epidemic strain, the vaccine efficacy declines [13].

IBV was considered to be less pathogenic than IAV. In most cases, IBV causes mild self-limiting respiratory infections. Nevertheless, many studies on IBV reported rates of hospitalization and deaths similar to IAV in children. Additionally, IBV infections in adults, although sporadic, have resulted in significant morbidity and mortality among the most vulnerable categories, hospital mortality being associated with higher heart rates, direct bilirubin levels, initial Pneumonia Severity Index (PSI) scores, and lower platelet levels [14].

Little is known about the epidemiology of influenza B in Egypt. The aim of this study was to describe the incidence and seroprevalence of IBV infections in Egypt between 2017 and 2019. This was performed through a household prospective cohort study that examined the incidence, human-to-human transmission, and prognosis of influenza infections among poultry-exposed growers in Egypt. Such information would assist public health authorities by understanding the burden of IBV in Egypt and hence issuing proper health policies to counter the effect.

## Materials and methods

### Ethics statement

Ethical approval for the study was granted by the IRBs of St. Jude Children’s Research Hospital (USA) and Human Link (Lebanon) as well as the Research Ethics Committee of the National Research Centre (Egypt). Written informed consent was obtained from all subjects over 18 years old, written assent was obtained for children between 14 and 17 years old, parental written consent was obtained for all participants less than 18 years old.

### Cohort study design

Details of the study design and protocol have been previously published [15]. Briefly, households raising backyard poultry were selected from five villages in northern Egypt starting August 2015. All individuals within the household who were older than two years were invited to participate. Baseline enrollment was completed in March 2017. A total of 2400 subjects were enrolled from 390 households in the five study sites. Study staff visited enrolled households on a weekly basis to check whether any study participant was reporting respiratory illness symptoms i.e. fever of 38 ˚C or higher and cough or sore throat. When a study participant was verified to have symptoms, a nasal swab and an oropharyngeal swab were obtained and tested by RT-PCR for influenza B infections. A serum sample was obtained from all participants on an annual basis regardless of symptom occurrence or disease seasonality (which follows the Northern Hemisphere).

### Viral testing

Swab samples were subjected to viral RNA (vRNA) extraction using QIAamp Viral-RNA Kit (Qiagen, Germany) according to manufacturer’s instructions. The vRNA extraction was followed by conventional RT-PCR using the OneStep RT‐PCR Kit (Qiagen). The vRNA was reverse transcribed and PCR-amplified in separate reactions. Briefly, 5 μl of vRNA extract were added to 5 μl of 5X PCR buffer, 1 μl dNTPs, 1 μl of enzyme mix, 1 μl forward and reverse Victoria lineage primers (0.5 μl from each primer) (Bvf224 ACATACCCTCGGCAAGAGTTTC and Bvr507 TGCTGTTTTGTTGTTGTCGTTTT), 1 μl forward and reverse Yamagata lineage primers (0.5 μl from each primer) (Byf 226 ACACCTTCTGCGAAAGCTTCA and Bvr63 CATAGAGGTTCTTCATTTGGGTTT), then the total volume of the reaction was adjusted to 25 μl using nuclease-free water. Samples were subjected to cDNA synthesis at 60°C/1 min, 42°C/20 min, and 50°C/20 min, followed by activation at 95°C/15 min, PCR amplification (35 cycles: 94°C/30 secs for denaturation, 52°C/30 secs for annealing, and 72°C/1 min for extension) and post-PCR extension at 72°C/10 min.

### Serological testing

Blood specimens were collected in vacuum tubes containing clotting agents. Clotted blood was kept on ice and delivered to the laboratory on the same day, where it was stored at 4°C. On the following day, serum was separated from cells by centrifugation for 5 minutes at 1000 xg and then aliquoted and frozen at −20°C until use. The microneutralization assay was used to measure the neutralizing antibody (nAb) titer in human sera. Briefly, the collected sera were inactivated at 56°C for 30 minutes. Sera were serially diluted two-fold from 1:40 to 1:5120 in Dulbecco’s Modified Eagle Medium (DMEM) (Gibco, Waltham, MA, USA) supplemented with 4% BSA (Sigma Aldrich, St. Louis, MO,USA), 2% antibiotic antimycotic mixture (Gibco), and 1 μg/ml TPCK-treated trypsin (Worthington Biochemical, Lakewood, NJ, USA), then mixed with equal volume of 100 tissue culture infectious dose (TCID50/mL) of either B/Brisbane/60/2008 (Victoria lineage) or B/Phucket/3073/2013 (Yamagata lineage) and incubated for 1 hr at 33°C. A total volume of 100 μl of the virus–sera mix was inoculated in duplicate to Madin-Darby canine kidney (MDCK) cells in a 96-well tissue culture plates. After 1 hr of incubation at 33°C, the inoculums were removed and 200 μl infection medium were added. The plates were then incubated for three more days at 33°C in 5% CO_2_ in a humidified incubator. A virus back-titration was performed without immune serum to confirm TCID50 viral titer used. Virus hemagglutination activity was then tested in 0.5% chicken red blood cells (RBCs). The absence of hemagglutination was considered a positive test result for antibodies to the virus. A titer of ≥40 was considered positive.

### Statistical analysis

Student’s t test was used to compare continuous variables and the Chi Square test was used to compare categorical variables. The McNemar test was used to compare seroprevalence and incidence accounting for repeated measurements. Logistic regression was used to determine factors affecting incidence or prevalence accounting for year and controlling for all other variables. The SPSS version 24 (IBM, Armonk, NY, USA) was used. A p-value <0.05 was considered statistically significant.

## Results

A total of 2400 subjects were enrolled in our study; the demographic distribution and health data of the study participants are shown in Table 1. Females constituted 54.9% of the study population. Around 44% were 18 years or younger. More than half of the participants (52%) had a primary and intermediate education, followed by those who were uneducated (34.3%), and secondary or university educated individuals (13.5%). Almost half of the subjects were single, and the rest were either married, divorced, or widowed. Students constituted 32.8%, housewives 29.2%, preschoolers (<6 years) 14.2%, and the rest were either professionals, skilled laborers, or unemployed. Most of the participants did not suffer from chronic diseases. The age range of the participants was 2 to 104 years old, and the mean age of the subjects was 26.65 years with standard deviation of 18.46 years.

**Table 1:** Distribution of demographic and health data of the study* participants, 2017–2019 in Egypt.

Variable	No. (%)
**Age**	
< 6 years	175 (7.3)
6–18 years	882 (36.8)
19–50 years	1026 (42.8)
> 51 years	314 (13.1)
**Sex**	
Female	1317 (54.9)
Male	1080 (45.1)
**Educational level**	
Not educated	822 (34.3)
Elementary/ Intermediate	1248 (52.1)
Secondary	130 (5.4)
College	194 (8.1)
**Marital Status**	
Single	1232 (51.4)
Married	1048 (43.7)
Widowed/Divorced	117 (4.9)
**Occupation**	
Pre-schooler	339 (14.2)
Student	783 (32.8)
Housewife	698 (29.2)
Unskilled labor/Unemployed	282 (11.8)
Skilled labor /professional	287 (12.0)
**Chronic disease** **	
Yes	250 (10.4)
No	2147 (89.6)

* Totals do not add up to 2400 due to missing data.

** Chronic diseases were chronic lung diseases, heart conditions, chronic kidney disease, and chronic liver disease.

Seroprevalence of antibodies against B/Brisbane/60/2008 (Victoria lineage) and B/Phuket/3073/2013 (Yamagata lineage) among study participants during the period from 2017 to 2020 is shown in Table 2. The overall seroprevalence among the 2400 participants, i.e., having at least one seropositive sample, was 53.2% against the Victoria lineage and 52.2% against the Yamagata lineage. During the 2017–2018 season, the total number of serum samples collected was 2280. The seroprevalence of Victoria antibodies was 13.9% and of Yamagata antibodies was 38.9%. In 2018–2019 season, 982 (49.4%) participants had antibodies against Victoria and 553 (27.7%) had antibodies against Yamagata from serum samples that were collected in this period. In the 2019–2020 season, the percent of seropositive subjects against Victoria and Yamagata was 30.4% and 9.9%, respectively. The difference of seroprevalence for either virus was statistically significant when years were compared (p<0.001). The difference of seroprevalence between viruses within the same season was also statistically significant (p<0.001). The detection of influenza B virus in participants who had ILI symptoms showed that 219 (9.1%) subjects were positive for influenza B virus during the 2017–2018 season and 183 (7.6%) subjects were positive during the 2018–2019 season by conventional RT-PCR. This difference between seasons was not statistically significant.

**Table 2:** Incidence and seroprevalence of Influenza B among study participants*, 2017–2019 in Egypt.

Year	No. [Positive %, (95% Confidence Interval)]		
	Victoria seroprevalence	Yamagata seroprevalence	Influenza B incidence
**2017–2018**	316/2280 [13.9 (12.5–15.4)]	886/2280 [38.9 (36.9–41.0)]	219/2400 [9.1 (8.0–10.4)]
**2018–2019**	982/1987 [49.4 (47.2–51.6)]	553/1998 [27.7 (25.7–29.7)]	183/2400 [7.6 (3.6–8.8)]
**2019–2020**	432/1423 [30.4 (28.0–32.8)]	141/1423 [9.9 (8.4–11.6)]	
**Overall**	1276/2400 [53.2 (51.2–55.2)]	1253/2400 [52.2 (50.2–54.2)]	

* Totals do not add up to 2400 due to missing data.

**Fig 1:**
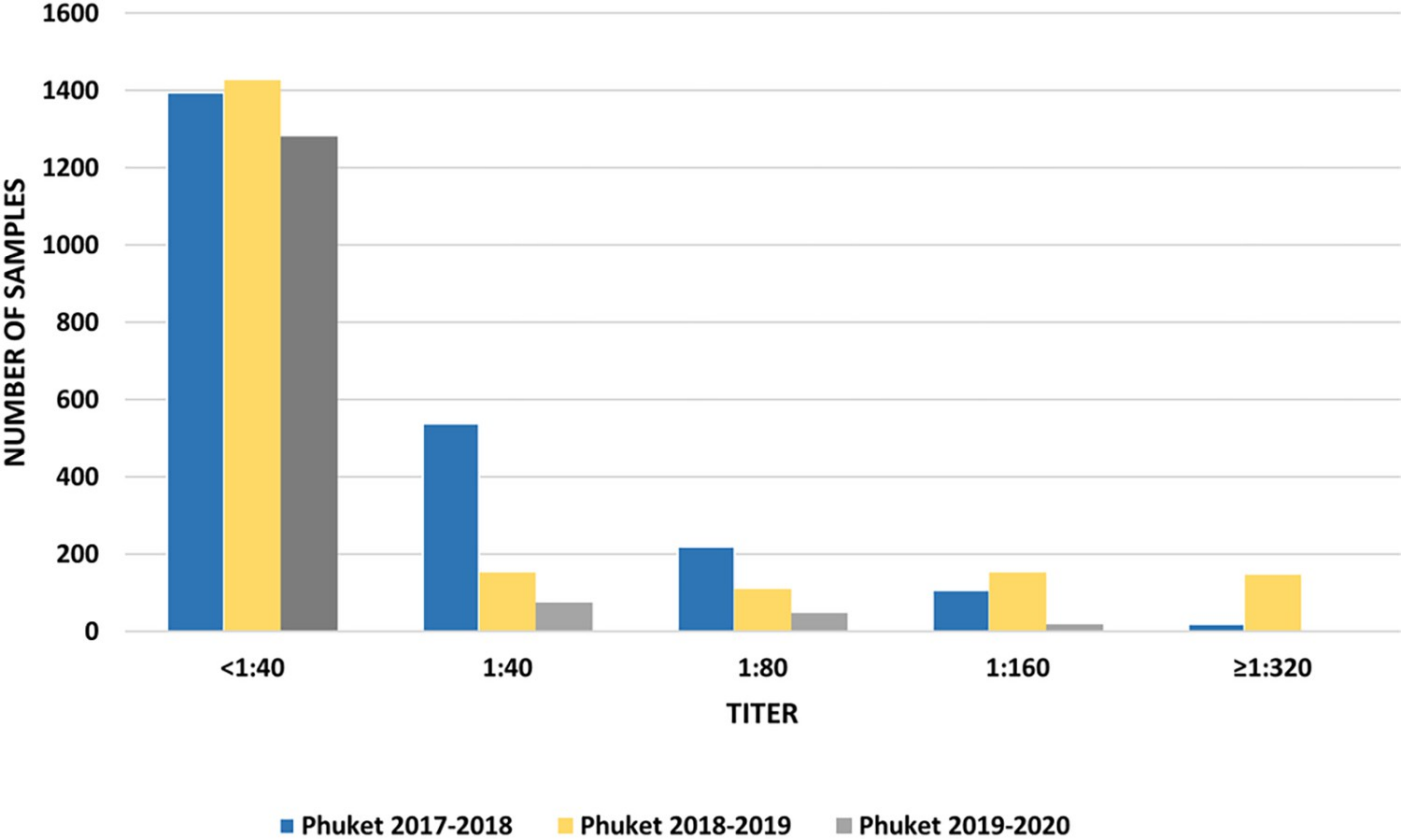
Titer distribution of antibodies against Yamagata lineage during each season of the study.

**Fig 2:**
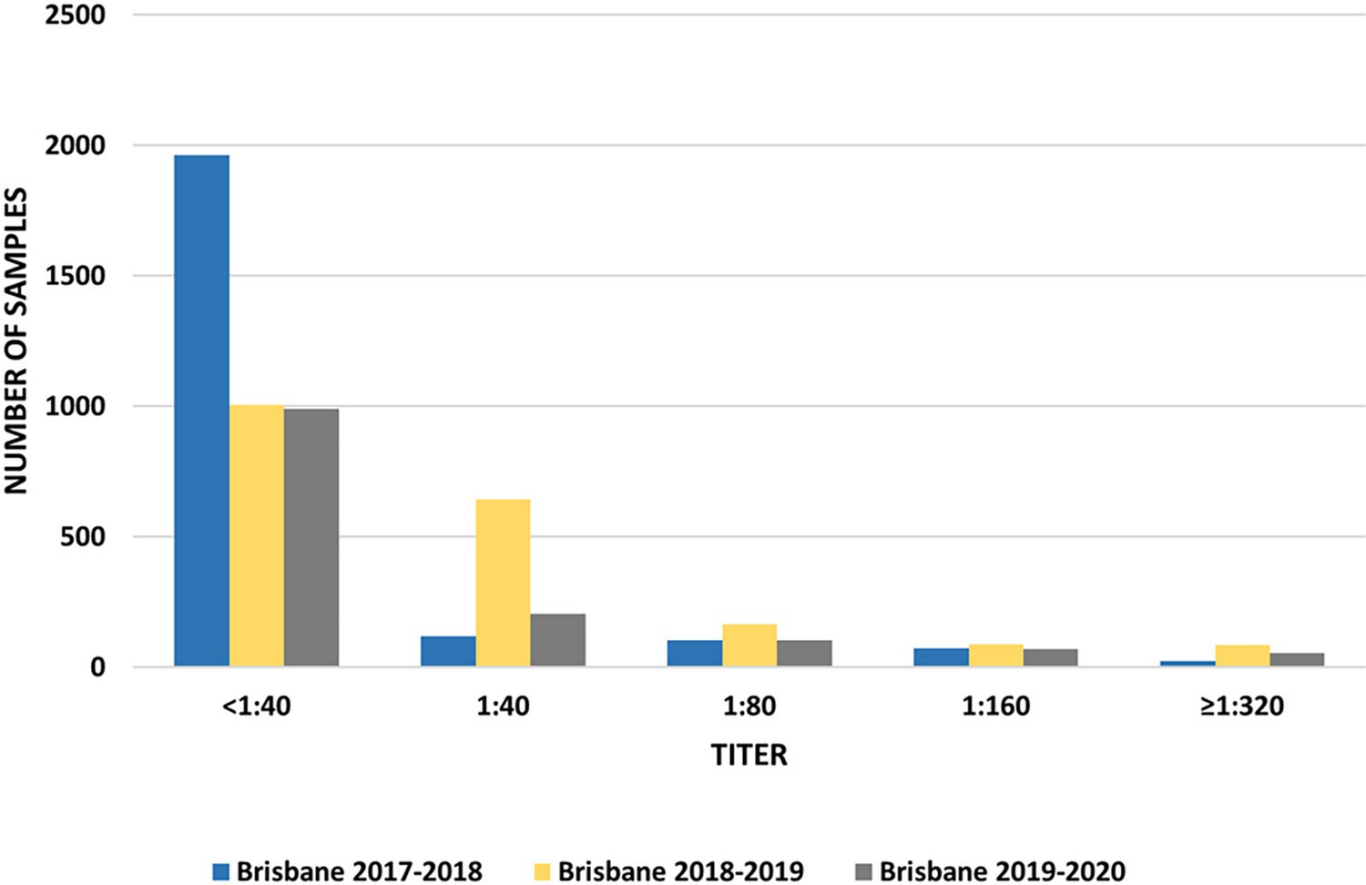
Titer distribution of antibodies against Victoria lineage during each season of the study.

Titer distribution is shown in Figs 1 and 2. The majority of the positive sera had a titer between 1:40 and 1:160.

During the 2017–2018 season, marital status, occupation, and age were associated with incidence of influenza B (Table 3). Most of the subjects who were positive were single (11.2%) and preschoolers (13.3%). The mean age of infected people was lower than non-infected people (p-value <0.001). During 2018–2019, gender was associated with incidence as females had a higher risk for getting the influenza B virus infection with an odds ratio of 1.4 and 95% confidence interval (1.1–1.9). Logistic regression showed that age and sex were the main predictor of IBV infection. The odds ratio of infection for children younger than 5 years was 1.34 (95% CI: 1.07–1.68).

**Table 3:** Determinants of incidence among study participants, 2017–2019 in Egypt.

**Incidence 2017–2018**			
**Variable**	Incidence No. (%)	p-value	Odds Ratio (95% Confidence Interval)
**Marital Status**			
Single	138 (11.2)	0.001	-
Married	75 (7.2)		
Widowed/Divorced	6 (5.1)		
**Occupation**			
Pre-schooler (2–5 years old)	45 (13.3)	<0.001	-
Student	86 (11.0)		
Housewife	55 (7.9)		
Unskilled labor/Unemployed	15 (5.3)		
Skilled labor /professional	17 (5.9)		
**Age**			
Mean age infected (SD)	21.4 (17.1)	<0.001	-
Mean age uninfected (SD)	27.2 (18.5)		
**Incidence 2018–2019**			
**Sex**			
Female	115 (8.7)	0.025	1.4 (1.1–1.9)
Male	68 (6.3)		

Education level, marital status, occupation, and age were associated with influenza B seroprevalence (Table 4). For Victoria lineage in seasons 2017–2018 and 2019–2020, the majority of seropositive participants had intermediate education, were single, and were students. The age range of seronegative people was higher than seropositive people (p-value = 0.001). In 2018–2019, single individuals and students constituted the large seropositive group among the study participants against Victoria lineage. The mean age of infected people was lower than non-infected people (p-value <0.001).

**Table 4:** Determinants of seroprevalence among study participants, 2017–2019 in Egypt.

Variable	Seropositive No. (%)	p-value
**Seroprevalence Victoria 2017–2018**		
**Educational level**		
Not educated	87 (11.6)	<0.001
Elementary/ Intermediate	201 (16.6)	
Secondary	12 (9.4)	
College	15 (8.1)	
**Marital Status**		
Single	199 (17.2)	<0.001
Married	104 (10.3)	
Widowed/Divorced	13 (11.4)	
**Occupation**		
Pre-schooler (2–5 years old)	27 (9.4)	<0.001
Student	163 (21.3)	
Housewife	69 (10.4)	
Unskilled labor/Unemployed	32 (11.7)	
Skilled labor /professional	24 (8.6)	
**Age**		
Mean age seropositive (SD)	22.7 (17.6)	<0.001
Mean age seronegative (SD)	27.7 (18.4)	
**Seroprevalence Victoria 2018–2019**		
**Marital Status**		
Single	579 (56.3)	<0.001
Married	361 (42.0)	
Widowed/Divorced	42 (43.8)	
**Occupation**		
Pre-schooler	162 (55.1)	<0.001
Student	378 (57.4)	
Housewife	236 (40.3)	
Unskilled labor/Unemployed	86 (41.0)	
Skilled labor/professional	117 (50.9)	
**Age**		
Mean age seropositive (SD)	24.0 (17.7)	<0.001
Mean age seronegative (SD)	28.4 (18.5)	
**Seroprevalence Victoria 2019–2020**		
**Educational level**		
Not educated	142 (30.0)	0.048
Elementary/ Intermediate	235 (32.6)	
Secondary	28 (29.8)	
College	27 (20.5)	
**Marital Status**		
Single	279 (38.1)	<0.001
Married	136 (22.2)	
Widowed/Divorced	17 (22.1)	
**Occupation**		
Pre-schooler	86 (41.1)	<0.001
Student	177 (37.3)	
Housewife	94 (22.3)	
Unskilled labor/Unemployed	30 (20.4)	
Skilled labor/professional	45 (27.4)	
**Age**		
Mean age seropositive (SD)	21.8 (16.9)	<0.001
Mean age seronegative (SD)	28.5 (18.5)	
**Seroprevalence Yamagata 2017–2018**		
**Marital Status**		
Single	482 (41.6)	0.009
Married	367 (36.5)	
Widowed/Divorced	35 (30.7)	
**Occupation**		
Pre-schooler	96 (33.6)	0.008
Student	334 (43.5)	
Housewife	249 (37.4)	
Unskilled labor/Unemployed	107 (39.2)	
Skilled labor/professional	95 (34.1)	
**Age**		
Mean age seropositive (SD)	25.5 (17.6)	<0.001
Mean age seronegative (SD)	27.9 (18.8)	
**Seroprevalence Yamagata 2018–2019**		
**Marital Status**		
Single	323 (31.2)	<0.001
Married	214 (24.7)	
Widowed/Divorced	16 (16.7)	
**Occupation**		
Pre-schooler	79 (26.8)	0.009
Student	215 (32.4)	
Housewife	153 (26.1)	
Unskilled labor/Unemployed	44 (20.8)	
Skilled labor/professional	61 (26.3)	
**Age**		
Mean age seropositive (SD)	23.2 (15.9)	<0.001
Mean age seronegative (SD)	27.3 (19.0)	
**Seroprevalence Yamagata 2019–2020**		
**Educational level**		
Not educated	24 (5.1)	<0.001
Elementary/ Intermediate	85 (11.8)	
Secondary	16 (17.0)	
College	16 (12.1)	
**Age**		
Mean age seropositive (SD)	22.6 (13.6)	0.008
Mean age seronegative (SD)	26.9 (18.7)	

As for the Yamagata lineage in 2017–2018 and 2018–2019, the majority of seropositive cases were students and unmarried participants. In 2019–2020, secondary-educated people were the most seropositive among study participants (17.0%) and the age range of the seronegative individuals was higher than the seropositive (p-value = 0.008).

Logistic regression showed that age and having chronic diseases were predictors of having IBV antibodies. The odds ratio for having IBV antibodies among children younger than 5 years was 1.44 (95%CI:1.23–1.69) and for those with chronic diseases was 1.48 (95%CI: 1.10–1.97).

## Discussion

Our study aimed to describe the incidence and seroprevalence, and to study the risk factors associated with influenza B infection in a rural population in Egypt between 2017–2020. Because of the limited data about the seroprevalence of the circulating influenza B in Egypt, there is a gap in the understanding of the epidemiology and burden of IBV disease.

The detection of IBV in the study participants with ILI showed that 9.1% of subjects were positive during the 2017–2018 season and that most of the subjects who were positive were single and preschoolers. Moreover, 7.6% of subjects were positive during the season of 2018–2019 and females had higher risk of infection than males. Detection rates of IBV infections varies between countries and over time. A virological surveillance study was conducted in Japan to detect and determine the genetic and antigenic characterization of IBV circulating during the 2017–2018 and 2018–2019 seasons, a total of 554 respiratory specimens were collected from ILI subjects in Tokyo, Japan out of which 108 samples were confirmed positive for influenza B (19.49%) [16]. In Lebanon, during the 2016–2017 influenza season, 519 nasopharyngeal swabs were collected from patients with ILI, 40.8% (212/519) were positive for influenza virus, 52% (110/212) were IAV and 48% (102/212) were IBV and this confirms that IBV has the same burden as IAV. During 2017–2018 season, among a total of 370 collected samples, 92 samples were influenza positive out of which 79.3 (73/92) were positive IAV and 20.6% (19/92) were IBV [17].

Several epidemiological studies conducted during influenza epidemics showed that influenza infection rates are higher among infants and children. In Germany, a total of 1,111 sera were collected from children and adolescents aged 0–18 years to determine the seroprevalence of influenza A and B antibodies using enzyme-linked immunosorbent assays. The results showed that, in children, the prevalence of antibodies against influenza B was 9.6% whereas in adults the prevalence of antibodies against influenza B was 56.7% [18].

Our serological findings show that the overall seroprevalence among the 2400 participants was 53.2% against the Victoria lineage and 52.2% against the Yamagata lineage, and by studying the determinants and risk factors for being seropositive against influenza B virus we found that, the majority of seropositive participants were school students where close contact increases the transmission of viral infection. This confirms that influenza B virus commonly affects children [19,20]. This potentially leads to high percentage of seropositivity among housewives, due to their constant contact with their children. This was also confirmed by our incidence data as the majority of cases were very young and the risk was higher in females. In Germany, a study done from 2008–2010 to determine influenza seroprevalence of children up to the age of 17 years showed that the overall prevalence was 47.0% in children and the prevalence of antibodies against influenza B increased significantly with age (p<0.001) [20]. In Serbia, a study in 2011–2013 showed that the prevalence of antibodies against influenza A and B viruses were not significantly different in the same year. The proportions of IgG-positive patients for influenza B in 2011 were 34.3% (93/271), in 2012 were 69.3% (194/280), and in 2013 were 85.4% (264/309) showing a significant increase over time (p-value <0.0001). The majority of subjects who were seropositive for IBVs were adults with age groups aged 30–64 and >65 years with a seropositivity rate of 48.7% and 60.8%, respectively [21].

Vaccination is the most effective method for prevention and control of influenza infection. The contribution of influenza B to the seasonal influenza burden differs each year. Although there are two distinct influenza B virus lineages, trivalent influenza vaccines contain antigens from only one [22]. We show here that in Egypt, both influenza B lineages circulated between 2017 and 2020 almost in equal proportion. Considering the low level of cross-protection provided by the immunization with a vaccine containing antigen from a single influenza B lineage, the need for the development of quadrivalent vaccines containing influenza A H1N1 and H3N2 antigens in addition to influenza B antigens from both lineages is highlighted [23–25].

Co-circulation of both influenza B virus lineages and mismatch between circulating influenza B and B strains selected for vaccines result in increased morbidity and mortality due to the absence of the other influenza B lineage from the trivalent seasonal vaccine. The importance of quadrivalent influenza vaccine resides in their ability to broaden the immune response and minimize the chance of a B-mismatched season [22]. Egypt imported the quadrivalent vaccine for the 2020–2021 and 2019–2020 seasons and the trivalent vaccine containing the Victoria-lineage strain before that.

This study has several limitations. The seroprevalence is likely underestimated as collection of samples wasn’t conducted only after or during the influenza season but was spread over the year. Furthermore, incidence of influenza B may be underestimated due to the potential of missing infected cases that did not meet the ILI criteria. The findings of this study may not be generalizable to the general population as the samples were collected only from five sites in Egypt and sampling was restricted to rural areas, nonetheless the age and sex distribution of the cohort resembled those of the general Egyptian population.

In Egypt, we recommend planning for annual influenza vaccination that covers all the country especially children with chronic pulmonary, cardiac, and immunosuppression. This is stressed as our multivariate analysis showed that age and having chronic illness were predictive of infection and of having antibodies against IBV. Seasonal influenza vaccine uptake may be increased by several interventions addressing knowledge and attitudes, access to vaccines, and adopting best implementation practices. Increasing vaccine uptake would be very helpful in preventing future cases of IBV hospitalizations.

## Conclusions

Results of this study revealed that the number of humans infected with IBVs is much larger than expected and this was evident in seroprevalence and circulation of the two lineages of influenza B virus in Egypt during three consecutive influenza seasons.

## References

[1] Krammer F, Smith GJD, Fouchier RAM, Peiris M, Kedzierska K, Doherty PC, et al. Influenza. Nat Rev Dis Primers. 2018;4(1):3. Epub 20180628. doi: 10.1038/s41572-018-0002-y ; PubMed Central PMCID: PMC7097467. 29955068 PMC7097467

[2] Laporte M, Stevaert A, Raeymaekers V, Boogaerts T, Nehlmeier I, Chiu W, et al. Hemagglutinin cleavability, acid stability, and temperature dependence optimize influenza B virus for replication in human airways. Journal of Virology. 2019;94(1):e01430–19. doi: 10.1128/JVI.01430-19 31597759 PMC6912116

[3] Monamele CG, Vernet MA, Njankouo MR, Kenmoe S, Schoenhals M, Yahaya AA, et al. Genetic characterization of influenza B virus in Cameroon and high frequency of reassortant strains. Journal of Medical Virology. 2018;90(12):1848–55. doi: 10.1002/jmv.25273 30036447

[4] Rota PA, Wallis TR, Harmon MW, Rota JS, Kendal AP, Nerome K. Cocirculation of two distinct evolutionary lineages of influenza type B virus since 1983. Virology. 1990;175(1):59–68. Epub 1990/03/01. doi: 10.1016/0042-6822(90)90186-u 2309452

[5] McCullers JA, Saito T, Iverson AR. Multiple genotypes of influenza B virus circulated between 1979 and 2003. J Virol. 2004;78(23):12817–28. Epub 2004/11/16. doi: 10.1128/JVI.78.23.12817-12828.2004 ; PubMed Central PMCID: PMC525012. 15542634 PMC525012

[6] Jumat MR, Sugrue RJ, Tan B-H. Genetic characterisation of influenza B viruses detected in Singapore, 2004 to 2009. BMC research notes. 2014;7(1):1–19. doi: 10.1186/1756-0500-7-863 25435177 PMC4265450

[7] Zhu D, Lok C, Chao S, Chen L, Li R, Zhao Z, et al. Detection and characterization of type B influenza virus from influenza-like illness cases during the 2017–2018 winter influenza season in Beijing, China. Arch Virol. 2019;164(4):995–1003. Epub 2019/02/08. doi: 10.1007/s00705-019-04160-w . 30729995

[8] Jennings L, Huang QS, Barr I, Lee PI, Kim WJ, Buchy P, et al. Literature review of the epidemiology of influenza B disease in 15 countries in the Asia‐Pacific region. Influenza and other respiratory viruses. 2018;12(3):383–411. doi: 10.1111/irv.12522 29127742 PMC5907823

[9] Zaraket H, Hurt AC, Clinch B, Barr I, Lee N. Burden of influenza B virus infection and considerations for clinical management. Antiviral Research. 2021;185:104970. doi: 10.1016/j.antiviral.2020.104970 33159999

[10] Rivas MJ, Alegretti M, Coppola L, Ramas V, Chiparelli H, Goni N. Epidemiology and Genetic Variability of Circulating Influenza B Viruses in Uruguay, 2012–2019. Microorganisms. 2020;8(4). Epub 2020/04/25. doi: 10.3390/microorganisms8040591 ; PubMed Central PMCID: PMC7232498. 32325860 PMC7232498

[11] Cohen AL, Hellferscee O, Pretorius M, Treurnicht F, Walaza S, Madhi S, et al. Epidemiology of influenza virus types and subtypes in South Africa, 2009–2012. Emerg Infect Dis. 2014;20(7):1162–9. Epub 2014/06/25. doi: 10.3201/eid2007.131869 ; PubMed Central PMCID: PMC4073865. 24960314 PMC4073865

[12] Seleka M, Treurnicht FK, Tempia S, Hellferscee O, Mtshali S, Cohen AL, et al. Epidemiology of influenza B/Yamagata and B/Victoria lineages in South Africa, 2005–2014. PLoS One. 2017;12(5):e0177655. Epub 2017/05/26. doi: 10.1371/journal.pone.0177655 ; PubMed Central PMCID: PMC5444647. 28542324 PMC5444647

[13] Ambrose CS, Levin MJ. The rationale for quadrivalent influenza vaccines. Hum Vaccin Immunother. 2012;8(1):81–8. Epub 2012/01/19. doi: 10.4161/hv.8.1.17623 ; PubMed Central PMCID: PMC3350141. 22252006 PMC3350141

[14] Liu D, Xu J, Yu X, Tong F, Walline J, Fu Y, et al. Clinical characteristics and prognosis of influenza B virus-related hospitalizations in northern China during the 2017–18 influenza season: a multicenter case series. BioMed research international. 2019;2019. doi: 10.1155/2019/8756563 31828141 PMC6885173

[15] El Rifay AS, Elabd MA, Abu Zeid D, Gomaa MR, Tang L, McKenzie PP, et al. Household Transmission of Zoonotic Influenza Viruses in a Cohort of Egyptian Poultry Growers. JMIR Res Protoc. 2015;4(2):e74. Epub 2015/06/24. doi: 10.2196/resprot.4331 ; PubMed Central PMCID: PMC4526956. 26099368 PMC4526956

[16] Kato‐Miyashita S, Sakai‐Tagawa Y, Yamashita M, Iwatsuki‐Horimoto K, Ito M, Tokita A, et al. Antigenic variants of influenza B viruses isolated in Japan during the 2017‐2018 and 2018‐2019 influenza seasons. Influenza and other respiratory viruses. 2020;14(3):311–9. doi: 10.1111/irv.12713 31955521 PMC7182600

[17] AlIbrahim M, Assaf-Casals A, Massaad E, Shaker R, Soudani N, Fayad D, et al. Molecular epidemiology and genetic characterization of influenza B virus in Lebanon during 2016–2018. Infection, Genetics and Evolution. 2019;75:103969. doi: 10.1016/j.meegid.2019.103969 31325610

[18] Sauerbrei A, Schmidt-Ott R, Hoyer H, Wutzler P. Seroprevalence of influenza A and B in German infants and adolescents. Medical microbiology and immunology. 2009;198(2):93–101. doi: 10.1007/s00430-009-0108-7 19194722

[19] Bhat YR. Influenza B infections in children: A review. World Journal of Clinical Pediatrics. 2020;9(3):44. doi: 10.5409/wjcp.v9.i3.44 33442534 PMC7769779

[20] Sauerbrei A, Langenhan T, Brandstädt A, Schmidt-Ott R, Krumbholz A, Girschick H, et al. Prevalence of antibodies against influenza A and B viruses in children in Germany, 2008 to 2010. Eurosurveillance. 2014;19(5):20687. doi: 10.2807/1560-7917.es2014.19.5.20687 24524235

[21] Radovanov J, Milošević V, Hrnjaković I, Petrović V, Ristić M, Elez I, et al. Influenza A and B Viruses in the population of Vojvodina, Serbia. Archives of Biological Sciences. 2014;66(1):43–50.

[22] Ray R, Dos Santos G, Buck PO, Claeys C, Matias G, Innis BL, et al. A review of the value of quadrivalent influenza vaccines and their potential contribution to influenza control. Human vaccines & immunotherapeutics. 2017;13(7):1640–52. doi: 10.1080/21645515.2017.1313375 28532276 PMC5512791

[23] Belshe RB. The need for quadrivalent vaccine against seasonal influenza. Vaccine. 2010;28:D45–D53. doi: 10.1016/j.vaccine.2010.08.028 20713260

[24] Weir JP, Gruber MF. An overview of the regulation of influenza vaccines in the United States. Influenza and other respiratory viruses. 2016;10(5):354–60. doi: 10.1111/irv.12383 27426005 PMC4947948

[25] Verma S, Soto J, Vasudevan A, Schmeisser F, Alvarado-Facundo E, Wang W, et al. Determination of influenza B identity and potency in quadrivalent inactivated influenza vaccines using lineage-specific monoclonal antibodies. PLoS One. 2017;12(4):e0175733. doi: 10.1371/journal.pone.0175733 28423025 PMC5396888

